# Comparative Study of Astaxanthin, Cholesterol, Fatty Acid Profiles, and Quality Indices Between Shrimp Oil Extracted From Hepatopancreas and Cephalothorax

**DOI:** 10.3389/fnut.2021.803664

**Published:** 2021-12-15

**Authors:** Navaneethan Raju, Saqib Gulzar, Natchaphol Buamard, Lukai Ma, Xiaoguo Ying, Bin Zhang, Soottawat Benjakul

**Affiliations:** ^1^International Center of Excellence in Seafood Science and Innovation, Faculty of Agro-Industry, Prince of Songkla University, Hat Yai, Thailand; ^2^Guangdong Provincial Key Laboratory of Lingnan Specialty Food Science and Technology, College of Light Industry and Food, Zhongkai University of Agriculture and Engineering, Guangzhou, China; ^3^Academy of Contemporary Agricultural Engineering Innovations, Zhongkai University of Agriculture and Engineering, Guangzhou, China; ^4^Zhejiang Provincial Key Laboratory of Health Risk Factors for Seafood, Collaborative Innovation Center of Seafood Deep Processing, College of Food and Pharmacy, Zhejiang Ocean University, Zhoushan, China; ^5^College of Biosystems Engineering and Food Science, Zhejiang University, Hangzhou, China; ^6^College of Food and Pharmacy, Zhejiang Ocean University, Zhoushan, China

**Keywords:** shrimp oil, nutrition indices, PUFA, astaxanthin, cholesterol

## Abstract

Shrimp oil from two different portions of Pacific white shrimp including cephalothorax and hepatopancreas was extracted using the mixture of hexane/isopropanol (1:1). The extracted oils from the cephalothorax (CPO) and hepatopancreas (HPO) were characterized for astaxanthin content, cholesterol levels, and fatty acid profiles. Nutrition indices of CPO and HPO were also compared. CPO had lower extraction yield (3.2 ± 0.1%, wet weight basis) than HPO (11.1 ± 0.5%, wet weight basis). High-performance liquid chromatography results indicated that the astaxanthin content in HPO was higher, compared to that of CPO. Nevertheless, the cholesterol level in HPO was 70% lower than that of CPO. Fatty acid profiles of HPO and CPO demonstrated that the polyunsaturated fatty acid (PUFA) content in HPO was higher than that of CPO. The amount of docosahexaenoic acid in the former was ~2 times higher than that of the latter. HPO contained 42.76 ± 0.36% PUFA, whereas PUFA content of CPO was 35.27 ± 0.19%. On the other hand, saturated fatty acids (SFA) were more pronounced in CPO (38.44 ± 0.26%) than HPO (30.82 ± 0.55%). Based on nutrition indices, namely, atherogenicity index, thrombogenicity index, hypocholesterolemic/hypercholesterolemic (h/H) ratio, and PUFA/SFA ratio, HPO possessed higher health benefit than CPO. The oxidation status of CPO and HPO measured in terms of peroxide value, thiobarbituric acid reactive substances, anisidine value, and conjugated dienes indicated that higher primary oxidation products were present in CPO, whereas HPO exhibited more secondary oxidation compounds. Fourier transform infrared spectra further substantiated the presence of oxidation products in CPO and HPO. Liquid chromatography-mass spectrometry identification showed the enhanced levels of phospholipids and glycolipids in the ethanolic fraction of CPO. Overall, HPO with a higher yield was more beneficial in terms of health benefits than CPO.

## Introduction

Increased shrimp demand, particularly in the forms of ready-to-cook or ready-to-eat, has led to an increasing amount of farmed shrimp (1). During the processing, byproducts, namely, cephalothorax, carapace, tail, and internal organs were produced and discarded (2). Apart from whole shrimp or peeled shrimp, some products with cephalothorax free of hepatopancreas are in demand in some markets. Thus, the hepatopancreas is removed using the sucking machine (3). As a consequence, hepatopancreas rich in oil can serve as a potential source for oil extraction (3). The cephalothorax has been prominently utilized for producing chitin, protein hydrolysate, and shrimp oil (4, 5). Concerning shrimp oil production, hepatopancreas having higher lipid content seems to render higher yield, compared to cephalothorax (3). Takeungwongtrakul et al. (3) reported that hepatopancreas has 11.79 ± 0.41% lipid content, while lipid content of 3.73 ± 0.34% was found in cephalothorax. In addition, the residues after oil extraction have become less when hepatopancreas is used, compared to the cephalothorax. Shrimp oil is rich in astaxanthin, astaxanthin esters, and polyunsaturated fatty acids (PUFAs), especially eicosapentaenoic acid (EPA) and docosahexaenoic acid (DHA) (6, 7). These bioactive compounds are highly beneficial for improving human health. Astaxanthin, a powerful antioxidant, possesses antiaging, anti-inflammation, and anticancer properties (5, 8–10). DHA and EPA have been known for improving brain and cardiac health (5, 11, 12). However, shrimp oil also contained saturated fatty acids (SFAs), which could lead to an increased low-density lipoprotein level and altered inflammatory response. In general, the limitation of SFAs in the diet is of concern about the ratio of unsaturated fatty acids (UFAs) to total fatty acids (13). Nutritive indices such as atherogenicity index (IA), thrombogenicity index (IT), hypocholesterolemic/hypercholesterolemic (h/H) ratio, and PUFA/SFA were computed for monitoring the quality of lipid (14). These indices could be employed for the shrimp oil to indicate nutritive value.

Although shrimp oil has a high content of highly beneficial bioactive compounds, it also contains cholesterol (6). Shrimp lipid extracted from cephalothorax consists of 65–70 mg cholesterol/100 g of lipid (5–7). Despite the need for some cholesterol to make hormones, vitamin D, etc., high levels of cholesterol can combine with other substances to form plaque in blood, causing atherosclerosis (15). To improve the nutrition quality of shrimp lipid extracted from cephalothorax, cholesterol has been lowered by saponin and beta-cyclodextrin (16–18). However, the cholesterol content in shrimp oil from the hepatopancreas has not been studied. The complete profiling of fatty acids, astaxanthin, cholesterol, and nutrition indices could be useful for the consumers to intake the shrimp oil for health benefits. To our knowledge, no aforementioned information for shrimp oils from both cephalothorax and hepatopancreas exists. Therefore, this study was aimed to characterize the oils extracted from cephalothorax and hepatopancreas of Pacific white shrimp and to compare both oils in terms of fatty acid content, astaxanthin content, cholesterol level, oxidation status, and nutritive indices.

## Materials and Methods

### Chemicals

All the chemicals used in the experiment were of analytical grade and purchased from Merck (Darmstadt, Germany). Pacific white shrimp (*Litopenaeus vannamei*) cephalothorax (shrimp head) and hepatopancreas were gifted from Sea Wealth Frozen Food Co., Ltd., Songkhla province, Thailand and Sea Fresh Industry Public Company, Pak Nam, Mueang Chumphon, Thailand, respectively.

### Extraction of Oil From Cephalothorax and Hepatopancreas

Shrimp oil was extracted using the hexane/isopropanol extraction method as detailed by Raju and Benjakul (6). First, cephalothorax and hepatopancreas were blended with a blender at high speed for 1 min to obtain a homogenous paste. The paste (100 g) was added to 500 ml of hexane/isopropanol mixture (1:1). The mixture was homogenized at 9,000 rpm for 3 min with the aid of an IKA Labortechnik homogenizer (Selangor, Malaysia). After homogenization, the solvent phase was separated by centrifugation using a Hitachi centrifuge (Hitachi Koki Co., Ltd, Tokyo, Japan) at 3,000 × *g* for 15 min at 4°C. The collected solvent phase was washed with an equal volume of distilled water and the process was repeated thrice. The hexane phase was collected and anhydrous sodium sulfate (10 g) was added to remove the water traces, followed by filtering using a Whatman filter paper No. 4 (Whatman International Ltd., Maidstone, England). The solvent was evaporated using an EYELA rotary evaporator N-1000 (Tokyo Rikakikai, Co., Ltd., Tokyo, Japan) at 40°C to obtain shrimp oil. Shrimp oil from cephalothorax and hepatopancreas were termed as “CPO” and “HPO,” respectively. Both the shrimp oils were flushed with nitrogen, placed in an amber vial, capped tightly, and stored at −40°C.

### Chemical Compositions, Nutritional, and Quality Indices of CPO and HPO

Both CPO and HPO were subjected to analyses.

#### Cholesterol and Astaxanthin Contents

The cholesterol and astaxanthin contents were quantified by high-performance liquid chromatography (HPLC) (Waters 2695 series, Milford, MA, USA) (6, 19) equipped with a reverse-phase Thermo scientific BDS-C18 column (5 μm; 150 × 4 mm) following the method of Raju et al. (20). Each oil sample (100 μl) was dissolved in 1 ml of ethanol. The mixture was vortexed vigorously for 1 min and stored at −18°C for 2 h. After incubation, the prepared mixtures were centrifuged at 3,600 × *g* for 10 min. A 100 μl of supernatant was taken and made up to 1 ml using mobile phase and injected in HPLC. HPLC program was performed at isocratic condition using 1.2 ml/min flow rate of methanol-acetonitrile (50:50) mixture as the mobile phase. A photodiode-array detector (Waters 2998, Milford, MA, USA) was used for the detection at 480 nm for astaxanthin and 202 nm for cholesterol. Authentic standards of astaxanthin (Dr. Ehrenstorfer GmbH, Augsburg, Germany) and cholesterol (Acros organics, Morris Plains, NJ, USA) were used for identification. The content was expressed as mg/g oil.

#### Fatty Acid Composition

Fatty acid profile was determined by the method of Raju et al. (20). Briefly, the sample (10 mg) was dissolved in 1 ml of hexane and esterified with 200 μl of 2 M methanolic sodium hydroxide at 50°C for 5 min. After cooling down, the mixture was vortexed and 200 μl of 2 M methanolic hydrochloric acid was added. The prepared mixture was vortexed thoroughly and then centrifuged at 3,500 × *g* for 10 min. The hexane phase was collected and injected into gas chromatography (Agilent GC 7890B; Santa Clara, CA, USA). Injection temperature was maintained at 250°C and the initial column temperature was first reduced to 80°C. The temperature was increased at 4°C min^−1^ for 40 min to 220°C and finally reached 240°C. The eluted compounds were identified by a flame ionization detector (Agilent GC 7890B; Santa Clara, CA, USA) at 270°C as a detector temperature. Genuine standards (Supelco FAME mix, Bellefonte, PA, USA) were used for the peak identification and the fatty acid content was expressed as a percentage.

#### Nutritional Indices

Nutritional indices were calculated based on the fatty acid profile by the following formulas (14).

The index of atherogenicity (IA) = [C12: 0 + (4 × C14: 0) + C16: 0] / [*Σ* MUFAs + *Σ* PUFAn6 + PUFAn3]

The index of thrombogenicity (IT) = [C14: 0 + C16: 0 + C18: 0]/ [(0.5 × *Σ* MUFA) + (0.5 × *Σ* ω-6 PUFA) + (3 × *Σ* ω-3 PUFA) + (ω-3/ω-6)]

The hypocholesterolemic/hypercholesterolemic (h/H) ratio = [cis C18: 1 + *Σ* PUFA]/ [C12: 0 + C14: 0 + C16: 0]

PUFA/SFA ratio = *Σ* PUFA/ *Σ* SFA

#### Lipid Oxidation

Both samples were determined for lipid oxidation using different indices.

##### Peroxide Value (PV)

Peroxide value (PV) was determined by the method of Pudtikajorn and Benjakul (21). An oil sample (0.1 g) was added with 25 ml of acetic acid/chloroform (3:2) mixture. To the prepared mixture, 1 ml of saturated potassium iodide was added and mixed. The mixture was then incubated in dark for 5 min. Subsequently, distilled water (75 ml) was added and shaken. At last, to the prepared mixture, 0.5 ml of 1% starch solution was added, shaken, and titrated with 0.01 N Na_2_S_2_O_3_. The titration was stopped after the disappearance of the dark blue color. PV was calculated and reported as mEq/ kg oil.

##### Thiobarbituric Acid Reactive Substances (TBARS)

Thiobarbituric acid reactive substances (TBARS) value was analyzed following the method of Gulzar and Benjakul (16). Quantification of the sample was done using the standard curve of 1,1,3,3-tetramethoxypropane (0–6 ppm) and the value was expressed as mg malonaldehyde/kg oil.

##### *p*-Anisidine Value (AnV)

*p*-Anisidine value (AnV) was measured as tailored by Firestone (22). Briefly, an oil sample (0.1 g) was mixed with 25 ml of isooctane and 0.5 ml of *p*-anisidine reagent. The absorbance of the mixture was read at 350 nm using a UV/vis spectrophotometer (Shimadzu UV-1800, Kyoto, Japan). AnV was calculated as follows:


$$\begin{array}{l} AnV=\;25\times \frac{\left(1.2\times A2\right)-A1}{W} \end{array}$$

where *A*1 and *A*2 are the absorbances before and after adding *p*-anisidine, respectively; *W* is the weight of the sample (g).

##### Conjugated Diene

Conjugated dienes (CDs) were analyzed using the method of Raju and Benjakul (17). Shrimp oil (0.1 g) was added to 100 ml of isooctane. The absorbance of the solution was read at 234 nm (*A*_234_). CD was calculated using the following equation:


$$\begin{array}{l} CD=\frac{A_{234}}{Weight\;of\;shrimp\;oil\;\left(g\right)\;\times \;cell\;path\;length\;\left(cm\right)} \end{array}$$

#### Fourier Transform Infrared (FTIR) Spectra

Fourier transform infrared (FTIR) spectra of samples were attained by Bruker Model Vector 33 FTIR spectrometer (Bruker Co., Ettlingen, Germany) following the method of Singh et al. (4). Wavenumber was selected between 4,000 and 500 cm^−1^ with 16 scans. A clean empty cell at 25°C was kept for normalization and as the reference background. The data were collected using OPUS 8.5 data collection software (Bruker Co. Billerica, MA, USA).

### LC-MS Identification of Ethanol-Soluble Lipids

Ethanol soluble lipids were prepared using the method of Raju et al. (18). Briefly, oil samples (100 mg) were added to 1 ml of ethanol and vortexed vigorously. The prepared mixture was then stored at −20°C for 3 h. At the end of the incubation period, the mixtures were centrifuged at 4,000 × *g* and the upper phase was collected. The upper phase (500 μl) was subjected to liquid chromatography quadrupole time-of-flight mass spectrometer (LC-QTOF MS), 1290 Infinity II LC-6545 Quadrupole-TOF (Agilent Santa Clara, CA, USA). The instrument was equipped with Zorbax Eclipse Plus C18 column (150 mm length × 2.1 mm inner-diameter, particle size 1.8 μm) (Agilent Santa Clara, CA, USA). Mobile phase A: 50 mM ammonium acetate/methanol [20/80 (v/v)] and B: Isopropanol/methanol [70/30 (v/v)] were used for the separation. Positive atmospheric pressure chemical ionization was done and compounds were identified based on mass and spectrum compared with MassHunter METLIN PCD library.

### Statistical Analysis

A completely randomized design was used for this study. Experiment and analysis were done in triplicate using three different sample lots. ANOVA was performed using the Statistical Package for Social Science SPSS software (IBM software, New York, NY, USA) (23). The *t*-test was used for pair comparison.

## Results and Discussion

### Chemical Compositions and Nutritional Indices of CPO and HPO

CPO and HPO had different yields. Yields of CPO and HPO were 3.2 ± 0.1% and 11.1 ± 0.5% (wet weight basis), respectively. Takeungwongtrakul et al. (3) reported that cephalothorax of Pacific white shrimp had a fat content of 3.73% (wet weight basis), while hepatopancreas consisted of 11.79% fat. The cephalothorax is the fused head and thorax including the outer shell (carapace), internal organs, and other components (24). The hepatopancreas is an important organ, which plays a major role in digestion and absorption (25). During the production of whole shrimp free of hepatopancreas, hepatopancreas was removed directly by a vacuum sucking machine (26). As a result, the obtained hepatopancreas was not adulterated by other organs, shells, or components (26). Hepatopancreas also functions as a lipid depositing organ (25). This was witnessed by four times higher fat content than that of cephalothorax.

#### Astaxanthin and Cholesterol

**Figure 1:**
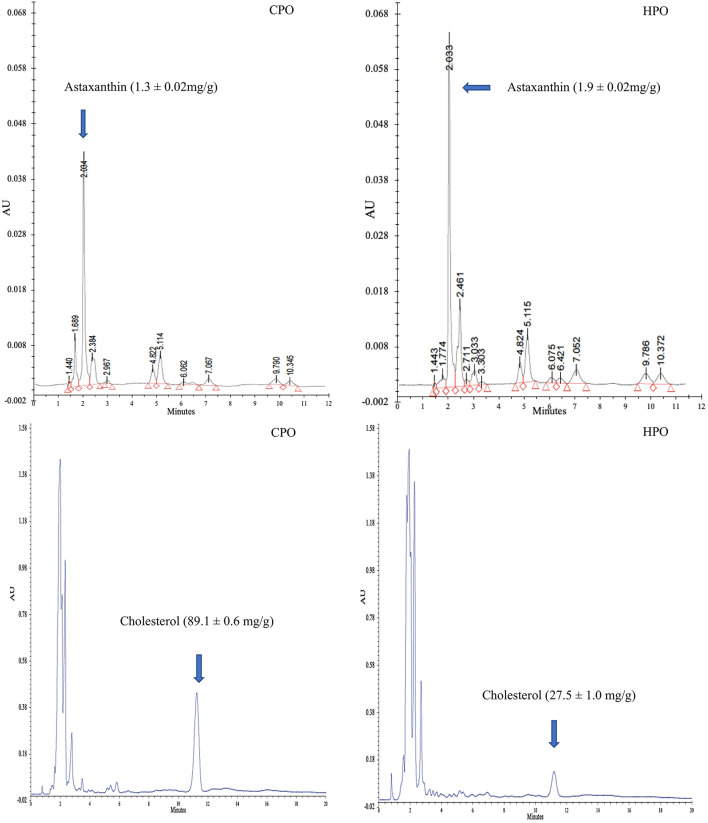
HPLC chromatogram of astaxanthin and cholesterol from CPO and HPO. CPO: Oil extracted from cephalothorax; HPO: oil extracted from hepatopancreas.

Chromatograms of astaxanthin and cholesterol in HPO and CPO are shown in Figure 1. Peak height and peak area have been used to quantify the target compounds. Higher astaxanthin content but lower cholesterol content was found in HPO than CPO (*p* < 0.05). Gómez-Estaca et al. (7) reported that oil from Pacific white shrimp cephalothorax had high cholesterol content (65 mg/g) but low astaxanthin content (7 mg/g). The hepatopancreas is a storage organ for carotenoids and lipids (25). As a principal carotenoid, astaxanthin plays a major role in photoprotection and as an antioxidant (27). For the biological response of crustaceans, astaxanthin is transported *via* hemolymph to encounter the response (28). As a primary carotenoid reservoir of the whole body (28), HPO contains high astaxanthin. However, as a principal sterol in crustaceans, cholesterol plays a vital role in maintaining cellular structure and controlling the rigidity of the membrane (29). Cholesterol maintains the membrane fluidity and protects crustaceans from salinity stress and temperature stress (29). Shrimps are bottom inhabitants and they survive in extreme environments (30). Cholesterol metabolism primarily takes place in the hepatopancreas (31) and is transported to the tissues for survivability and the control of stress (32) and to the ovaries for yolk development (31). Due to this reason, HPO contains lower cholesterol than CPO. In terms of health benefits, dietary cholesterol intake will lead to an increase in serum cholesterol levels and cardiovascular diseases (5). When comparing HPO and CPO, the former could provide more health benefits than the latter. Moreover, the augmented astaxanthin content would increase antioxidant activity, inflammatory protection, anticancer, and antiaging properties (5).

#### Fatty Acid Profile

Table 1 illustrates the fatty acid profile of CPO and HPO. Palmitic acid (SFA) was found to be the most dominant fatty acid in both oils. However, the second-highest fatty acid in HPO was DHA (22:6, n3) at 16.25%, which was higher than that of CPO (8.98%). The second highest fatty acid in CPO was linoleic acid (18:2, n-6), which constituted 14.22%. On the other hand, n-6 fatty acid content was higher in the latter (*p* < 0.05). For oleic acid (18:1, n-9), CPO showed higher content (13.18%) than HPO (9.87%) (*p* < 0.05). The total SFA and MUFA contents were higher in CPO. However, the health-beneficial PUFA content was higher in HPO. In general, n-3 fatty acid content was greater in HPO than CPO (*p* < 0.05). These results were in accordance with Gómez-Estaca et al. (7), Gulzar and Benjakul (16) who stated that palmitic acid was dominant in shrimp oil extracted from cephalothorax. Takeungwongtrakul et al. (3) documented that lipid from hepatopancreas contained lesser SFA content than that extracted from cephalothorax. Gómez-Estaca et al. (7) reported higher n-6 fatty acid than n-3 fatty acid in CPO from Pacific white shrimp. Increased n-6 fatty acid consumption has been known to produce eicosanoids that cause inflammation, while n-3 fatty acids show anti-inflammatory activity (33). Overall HPO contained fatty acids with health benefits to a higher extent, compared to CPO, mainly due to the presence of n-3 fatty acids, especially EPA and DHA.

**Table 1:** Fatty acid profiles of shrimp oil extracted from cephalothorax and hepatopancreas.

**Fatty acids (%)**	**CPO**	**HPO**
C12:0	0.74 ± 0.04a	0.31 ± 0.01b
C14:0	0.48 ± 0.01b	1.17 ± 0.05a
C15:0	0.33 ± 0.01b	0.80 ± 0.01a
C15:1	0.35 ± 0.01a	ND
C16:0	18.97 ± 0.36b	19.27 ± 0.61a
C16:1	1.10 ± 0.01b	2.23 ± 0.02a
C17:0	0.80 ± 0.01a	0.45 ± 0.04b
C17:1	0.50 ± 0.00	ND
C18:0	8.67 ± 0.05a	1.95 ± 0.04b
C18:1 n-9c	13.18 ± 0.17a	9.87 ± 0.03b
C18:2 n-3c	14.22 ± 0.14a	13.13 ± 0.02b
C20:0	0.46 ± 0.01	ND
C20:1	1.05 ± 0.07b	1.77 ± 0.14a
C18:3 n-3c	0.43 ± 0.01b	0.60 ± 0.00a
C20:2 n-6c	2.22 ± 0.02b	2.85 ± 0.04a
C20:0	0.76 ± 0.02	ND ± 0.00
C23:0	6.42 ± 0.03b	7.19 ± 0.03a
C24:0	0.81 ± 0.02	ND ± 0.00
C20:5 n-3c (EPA)	9.42 ± 0.02b	9.94 ± 0.06a
C24:1	1.06 ± 0.11	ND ± 0.00
C22:6 n-3c (DHA)	8.98 ± 0.03b	16.25 ± 0.27a
Others	9.06 ± 0.12b	12.25 ± 0.96a
Saturated Fatty Acid (SFA)	38.44 ± 0.26a	30.82 ± 0.55b
Monounsaturated Fatty Acid (MUFA)	17.24 ± 0.01a	13.86 ± 0.15b
Polyunsaturated Fatty Acid (PUFA)	35.27 ± 0.19b	42.76 ± 0.36a
n-3 Fatty acids	18.83 ± 0.05b	26.79 ± 0.32a
n-6 Fatty acids	16.44 ± 0.15a	15.98 ± 0.05b

*CPO, Oil extracted from cephalothorax; HPO, oil extracted from hepatopancreas; EPA, eicosapentaenoic acid; DHA, docosahexaenoic acid*.

*Different lowercase letters within the same row denote significant difference (p < 0.05)*.

#### Nutrition Indices

**Figure 2:**
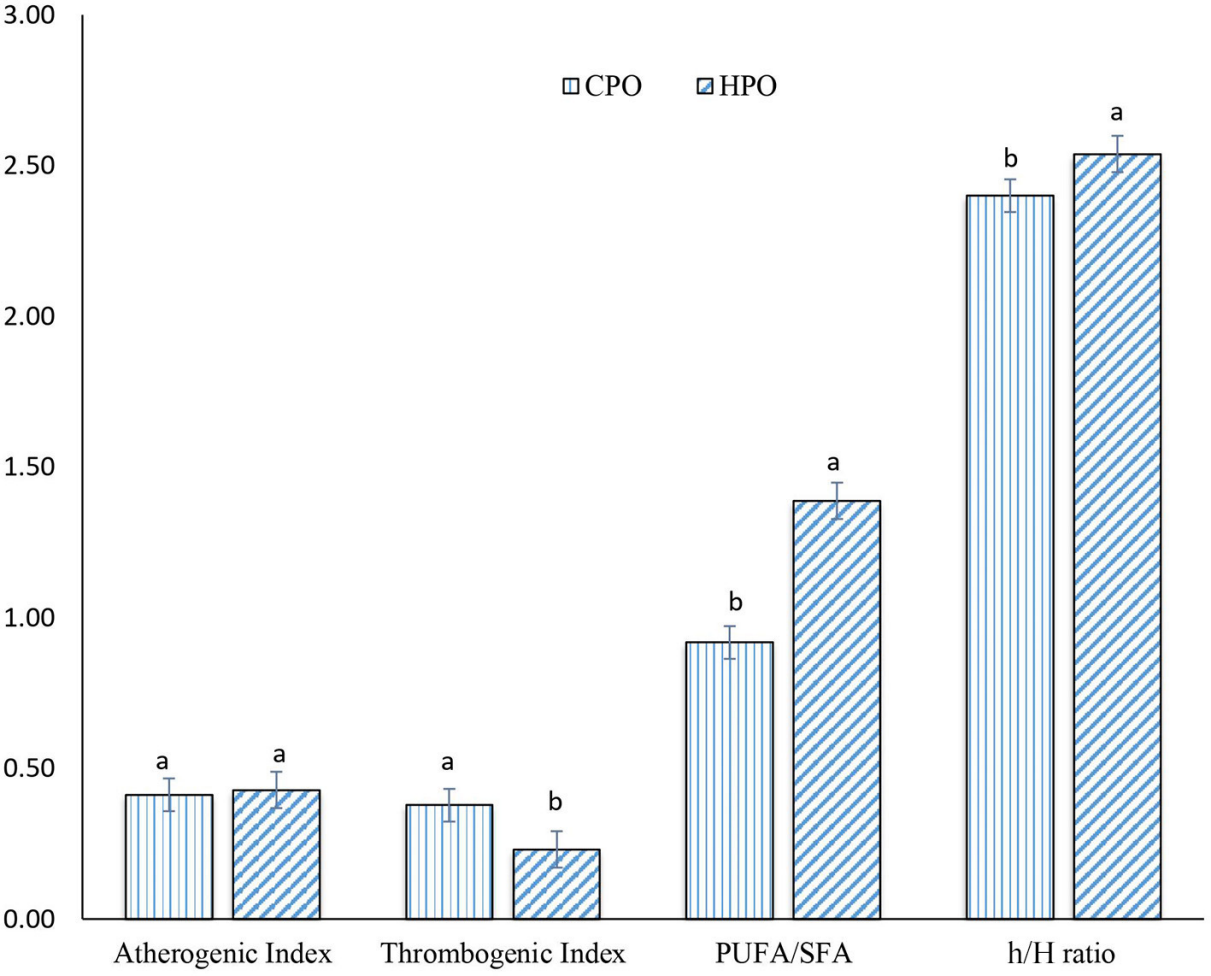
Nutrition indices of CPO and HPO. CPO: Oil extracted from cephalothorax; HPO: oil extracted from hepatopancreas. Different lowercase letters within the same nutrition index denote significant difference (*p* < 0.05).

Dietary oils extracted from plants and animals are composed of numerous fatty acids, classified as SFA, MUFA, and PUFA. However, the health benefits of the extracted oil are governed by the presence of beneficial PUFA with a low ratio of SFA (14). From fatty acid composition, nutrition indices were calculated to reveal the health-promoting index. Commonly used nutritive indices are IA, IT, and h/H (14). Apart from these, several indices such as PUFA/SFA, EPA + DHA, *trans* fatty acid, and unsaturation index have been also used to indicate the health benefit (13). In this current study, IA, IT, h/H, and PUFA/SFA were computed for both CPO and HPO (Figure 2).

A similar IA index was observed between CPO and HPO (*p* > 0.05). However, IT was lower in HPO, compared to that of CPO. Ulbricht and Southgate (34) developed and proposed IA and IT for calculating atherogenicity and thrombogenicity (14, 34). Atherogenicity for foods is calculated based upon SFA and UFA contents (35). Lauric acid (C12: 0), myristic acid (C14: 0), and palmitic acid (C16: 0) are considered to be atherogenic (35). These fatty acids can bind with the cells of the circulatory system, leading to the formation of plague (14). However, UFAs possess antiatherogenic activity, thus inhibiting plaque formation. Thus, IA was calculated from the ratio of proatherogenic SFAs to antiatherogenic UFAs (14). Thrombogenicity refers to the ratio of fatty acids inducing clot formation (C12:0, C14:0, and C16:0) and the fatty acids having antithrombotic effects such as MUFA, n-6, and n-3 fatty acids (34). Since HPO contained a higher amount of n-3 fatty acids, compared to CPO, this resulted in the lower IT in HPO. IA and IT have been considered as markers for assessing cardiovascular health (35). When comparing both the oils, HPO was considered as the better oil in terms of protecting heart diseases with higher anticlotting ability.

The h/H ratio was first developed in lamb meat by Santos-Silva et al. (36). The h/H ratio was found to be upgraded from PUFA/SFA ratio by selecting only three SFA (C12:0, C14: 0, and C16:0) that can induce hypercholesterolemic conditions rather than selecting total SFA. High h/H ratio and PUFA/SFA ratio are considered as health benefits in neutralizing hypercholesterolemic conditions mainly by PUFA (35). From a nutritional point of view, the h/H ratio between 0.5 and 1 is considered as an ideal value (37). According to the Dietary Guidelines for Americans and the European Society of Cardiology, the upper limit for the intake of total SFA is set at 7–10% of total energy intake, whereas the upper limit for consumption of PUFA is set between 6 and 11% of total energy intake (38). HPO possessed a higher h/H ratio and PUFA/SFA ratio. The augmented PUFA content in HPO led to a profound hypocholesterolemic effect as witnessed by an increased h/H ratio.

#### Lipid Oxidation

**Figure 3:**
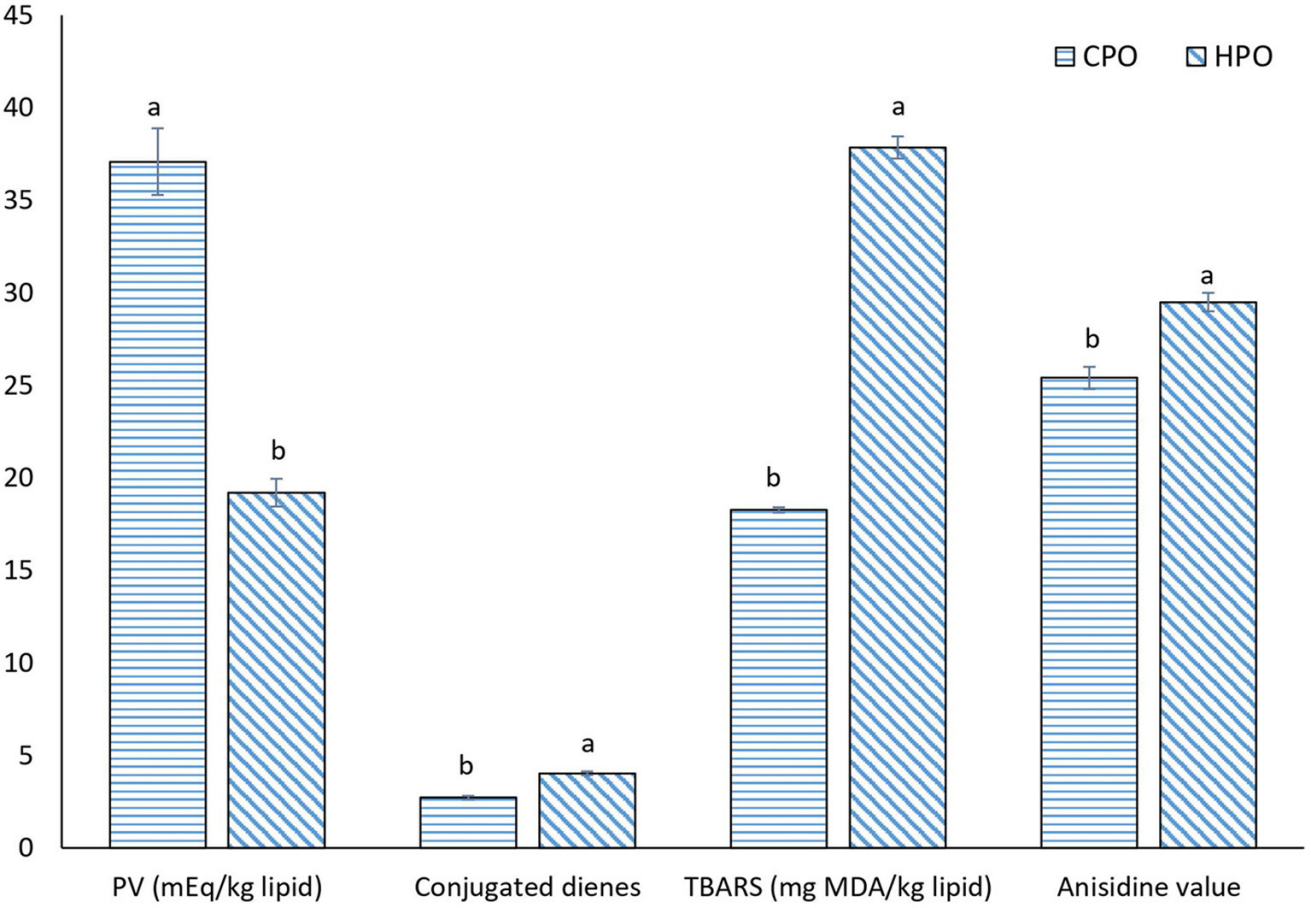
Oxidation status of CPO and HPO. CPO: Oil extracted from cephalothorax; HPO: oil extracted from hepatopancreas. Different lowercase letters within the same oxidation index denote significant difference (*p* < 0.05).

Lipid oxidation was assayed by measuring primary and secondary lipid oxidation products (Figure 3). PV has been used to monitor the formation of primary oxidation products, mainly hydroperoxides in the presence of O_2_. The formed hydroperoxides are not stable and more likely decomposed to secondary oxidation products such as aldehydes and ketones (3). PV was higher in CPO than HPO. For CDs, a higher CD was observed in HPO than CPO. CDs are also primary oxidation products. Lipid peroxidation starts with the abstraction of H from the -CH_2_- group of PUFAs. As a result, carbon radical is stabilized by a molecular rearrangement, forming CDs in which, two double bonds are separated by a single bond (39). With a high content of PUFA, HPO could undergo a higher abstraction of H, leading to the higher formation of CD. CDs were therefore defined as diene formation due to the presence of double bonds (21). However, the secondary oxidation products as indicated by TBARS value and AnV were higher in HPO. HPO was composed of higher PUFAs that were vulnerable to oxidation. At the beginning stage, primary oxidation products such as hydroperoxides were formed. Simultaneously, the decomposition occurred at a higher rate. As a result, the amount of hydroperoxide was decreased as indicated by lowered PV, while TBARS value and AnV were augmented. TBARS value has been widely used to measure volatile oxidation products, while AnV has been employed to measure non-volatile lipid oxidation products (3). AnV determines the amount of aldehydes, principally 2-alkenals and 2, 4-dienals by reaction with *p*-anisidine (40). The results were in accordance with Gulzar and Benjakul (16) who found that the oxidation of shrimp oil from cephalothorax was enhanced during the storage period, while the addition of antioxidants could suppress the oxidation. Overall, HPO with high PUFA content was prone to oxidation than CPO containing a lower amount of PUFA (Table 1).

#### FTIR Spectra

**Figure 4:**
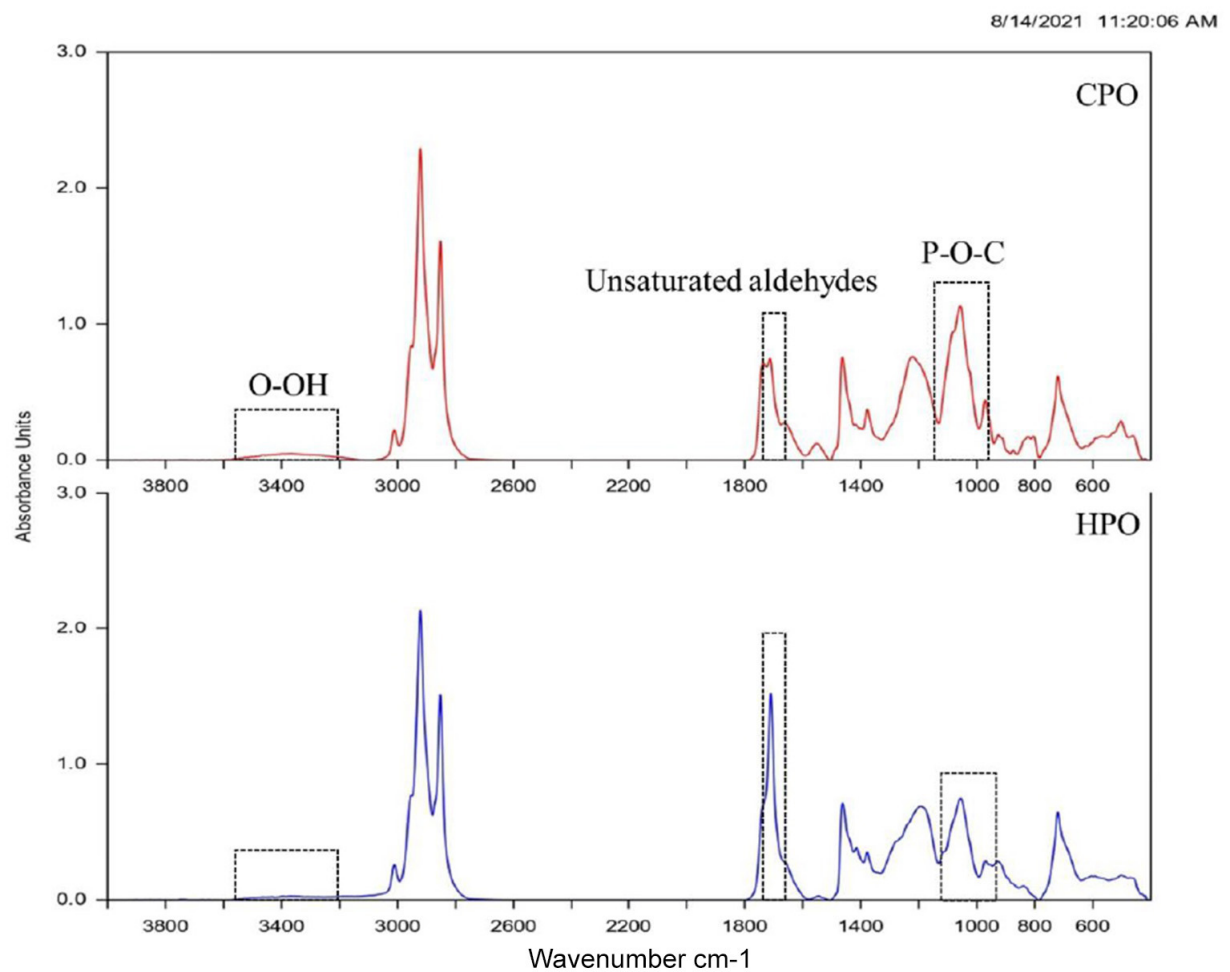
FTIR spectra of CPO and HPO. CPO: Oil extracted from cephalothorax; HPO: oil extracted from hepatopancreas.

Fourier transform infrared (FTIR) spectrum has been used for the identification of functional groups (41). Oil samples with the altered functional group formed due to oxidation could be identified *via* spectra (Figure 4). The wavenumber starting from 4,000 to 400 cm^−1^ was analyzed. The initial peak was found at 3,600–3,400 cm^−1^ in CPO and HPO, representing the O-OH group (16). This denotes the presence of the primary oxidation product of peroxide, mainly hydroperoxide. However, the O-OH peak was much lowered in HPO, representing the lower amount of peroxide formed. These results were in line with the PV result (Figure 3), in which higher PV was obtained in CPO than HPO. Other notable changes were found at 1,730–1,685 cm^−1^ and 1,200–970 cm^−1^, representing the unsaturated aldehydes (42) and P-O-C (43), respectively. Similar peaks representing lipid oxidation in shrimp oil extracted by ultrasonic-assisted extraction were posted by Gulzar and Benjakul (44). The peak height of aldehydes was greater in HPO, compared to that of CPO, reflecting the augmented formation of secondary lipid oxidation products in HPO. Phosphate attached with the carbonyl group indicates the presence of higher phospholipids in CPO (6). However, the peak was lowered in HPO, indicating the lower phospholipid content. Overall, FTIR spectra revealed higher aldehydes, lower peroxide, and phospholipid in HPO than CPO.

#### LC-MS Identification of Ethanol-Soluble Lipids

Ethanol soluble lipids were prepared by the ethanol crystallization method (18). The ethanol-soluble polar and neutral lipids such as cholesterol, astaxanthin, fat-soluble vitamins, phospholipids, and other carotenoids were separated from insoluble non-polar lipids. The non-polar lipids mainly triglycerides were crystallized due to the insolubility in ethanol (6). In the current study, preliminary identification of lipid components in the ethanolic fraction was done by liquid chromatography-mass spectrometry (LC-MS) (Table 2). When comparing both the samples, CPO was dominated by glycolipids, phospholipids, diacylglycerol, monoacylglycerol, and sterol derivatives. In HPO, diacylglycerol, monoacylglycerol, and sterol derivatives were predominantly found. Although HPO contained phospholipids as one of the major components, some phospholipids were not identified, compared to those found in CPO. The result showed that the phospholipids in HPO might be lower, compared to that of CPO. FTIR results (Figure 4) supported the LC-MS data by showing the smaller peak of phospholipid in HPO. Glycolipids were dominant in CPO but they were not detected in HPO. Glycolipids and phospholipids are the major components in cell membrane regulating osmoregulatory changes in crustacean cell membrane structures (45). Cephalothorax contains more membranes or tissues, compared to hepatopancreas. The presence of lesser tissue in the hepatopancreas was due to the target removal of the gland by a sucking machine (26). This could be the reason for fewer phospholipids and unidentified glycolipids. Phospholipids in both oils mainly consisted of phosphatidylethanolamine (PE) conjugated with different fatty acids. In general, shrimp lipid contains PE and phosphatidylcholine as major phospholipids (13). However, the lipid components can vary due to seasonal changes, diet, maturity, and size (5). C30 column was used for the identification of astaxanthin esters and other neutral lipids in shrimp lipid (7), whereas C18 was the column used in the study. This could lead to the difference in profiles of components in oils. Other components found in both the samples were sterol derivatives, vitamin derivatives, α-carotene, and astaxanthin. Based on the HPLC result, higher astaxanthin was found in HPO, thus providing more health benefits than CPO. Overall, the LC-MS identification revealed that HPO also contained vitamins with lesser phospholipid and higher astaxanthin contents.

**Table 2:** LC-MS identification of shrimp oil extracted from cephalothorax and hepatopancreas.

**Compound name**	**CPO**	**HPO**	**(M+H)+**	**(M+NH4)+**	**Formula**
	**Abundance**				
**Mono and Diacylglycerols**					
MG(18:0e/0:0/0:0)	33,405	9,184		344.3289	C21 H44 O3
MG(20:5(5Z,8Z,11Z,14Z,17Z)/0:0/0:0)	3,081	22,103	376.2628		C23 H36 O4
MG(22:2(13Z,16Z)/0:0/0:0)	ND	13,589		410.3399	C25 H46 O4
DG(14:1(9Z)/14:1(9Z)/0:0)	ND	1,012		508.4129	C31 H56 O5
DG(16:0e/18:0/0:0)	30,561	28,386	582.5586		C37 H74 O4
DG(19:1(9Z)/19:0/0:0)	ND	17,162		650.5852	C41 H78 O5
DG(20:3(8Z,11Z,14Z)/14:0/0:0)	ND	6,321	590.4918		C37 H66 O5
DG(22:5(7Z,10Z,13Z,16Z,19Z)/14:0/0:0)	1,139	1,019		614.4916	C39 H66 O5
DG(22:6(4Z,7Z,10Z,13Z,16Z,19Z)/16:0/0:0)	2,151	1,510		638.4917	C41 H66 O5
**Vitamin derivatives**					
26,27-Dihomo-1α-hydroxyvitamin D2	ND	11,283		440.3651	C30 H48 O2
Previtamin D3	ND	21,412		384.3394	
Vitamin E succinate	ND	4,274		530.3974	C33 H54 O5
3-Deoxyvitamin D3	5,120	29,595	368.3457		C27 H44
1α-butyl-1β,25- dihydroxycholecalciferol	3,192	4,723		472.3923	C31 H52 O3
**Wax esters**					
Linolenyl laurate	ND	1,051		446.4133	C30 H54 O2
Myristoleyl linolenate	ND	49,763		472.4287	C32 H56 O2
Cohibin D	167,231	24,795	548.4811		C37 H68 O4
Montecristin	23,185	303,785	577.5196		C37 H66 O4
Artemoin A	1,491	1,521	550.4985		C35 H66 O4
**Glycolipids**					
GlcCer(d14:2(4E,6E)/18:0)	11,258	ND	669.5173		C38 H71 N O8
GlcCer(d14:1/18:0)	4,011	ND	671.5329		C38 H73 N O8
GlcCer(d16:1/20:0)	42,315	ND	727.5948		C42 H81 N O8
GlcCer(d18:0/14:0)	1,018	ND	673.5483		C38 H75 N O8
GalCer(d18:0/16:0)	45,499	ND	701.5798		C40 H79 N O8
GlcCer(d18:0/18:0)	218,624	ND	729.6119		C42 H83 N O8
**Pigments**					
4,4'-Diapo-zeta-carotene	ND	1,210		404.3444	C30 H44
Persicaxanthin	ND	2,683	384.2666		C25 H36 O3
β-Carotene	1,098	1,202	536.4383		C40 H56
Astaxanthin	25,172	54,872	596.3851		C40 H52 O4
**Sterols**					
17 beta-Hydroxy-2alpha-(methoxymethyl)-17-methyl-5alpha-androstan-3-one	ND	76,329	348.2668		C22 H36 O3
Cholesterol	69,621	10,129	387.3621		C27H46O
Cystosterol	ND	21,412	384.34		C27 H44 O
Conicasterol D	5,044	9,135	444.3605	677.5362	C29 H48 O3
Cholest-5-ene	ND	29,595	370.3607		C27 H46
4,4,14α-trimethyl-5α-cholest-9 (11),24-dien-3β-ol	1,011	ND	426.3855		C30 H50 O
4α-methyl-24-methylene-cholestan-3β,8β,11β-triol	13,100	ND	446.3757		C29 H50 O3
4α-formyl-4β-methyl-5α-cholesta-8-en-3β-ol	63,909	66,326	428.3647		C29 H48 O2
**Phospholipids**					
PE(14:0/22:5(4Z,7Z,10Z,13Z,16Z))	1,129	ND	737.4995		C41 H72 N O8 P
PE(16:1(9Z)/P-18:1(11Z))	1,098	1,521	699.5207		C39 H74 N O7 P
PE(16:0/22:6(4Z,7Z,10Z,13Z,16Z,19Z))	21,319	ND	763.516		C43 H74 N O8 P
PE(O- 16:0/22:6(4Z,7Z,10Z,13Z,16Z,19 Z))	1,091	1,158	749.5372		C43 H76 N O7 P
PE(16:0/18:2(9Z,12Z))	1,519	ND	715.5143		C39 H74 N O8 P
PE(17:1(9Z)/22:6(4Z,7Z,10Z,13Z,16Z,19Z))	7,689	ND	775.514		C44 H74 N O8 P
PE(P-18:1(9Z)/18:4(6Z,9Z,12Z,15Z))	5,135	ND	721.5054		C41 H72 N O7 P
PE(P-18:1(9Z)/18:4(6Z,9Z,12Z,15Z))	ND	1,195	721.5073		C41 H72 N O7 P
PE(P-18:1(9Z)/20:5(5Z,8Z,11Z,14Z,17Z))	ND	1,152	747.5221		C43 H74 N O7 P
PE(18:1(9E)/18:1(9E))	14,047	ND		743.5475	C41 H78 N O8 P
PE(18:3(6Z,9Z,12Z)/P-18:1(11Z))	42,315	2,515	723.5211		C41 H74 N O7 P
PE(P-18:1(9Z)/18:4(6Z,9Z,12Z,15Z))	ND	1,195	721.5073		C41 H72 N O7 P
PE(22:6(4Z,7Z,10Z,13Z,16Z,19Z)/P-18:1(11Z))	ND	1,591	773.5357		C45 H76 N O7 P
PE(20:4(5Z,8Z,11Z,14Z)/20:4(8Z,11Z,14Z,17Z))	1,989	ND	787.5151		C45 H74 N O8 P
1-Alkyl-2-acylglycerophosphoethanolamine	10,986	5,186	751.554		C43 H78 N O7 P

*LC-MS, Liquid chromatography-mass spectrometry; CPO, oil extracted from cephalothorax; HPO, oil extracted from hepatopancreas; ND, not detected; PE, Phosphatidylethanolamine; MG, Monoacylglycerol; DG, Diacylglycerol; GlcCer, Glycolipid*.

## Conclusion

Oil extracted from cephalothorax and hepatopancreas of Pacific white shrimp showed different compositions and nutrition indices. Oil from the hepatopancreas with higher yields had higher amounts of astaxanthin and lower cholesterol levels. PUFAs were more pronounced in HPO. Consequently, HPO possessed the superior nutritive value to CPO. However, secondary oxidation products were found in HPO, indicating that lipid oxidation took place. Nevertheless, the oxidation in both the samples could be overcome with the addition of natural antioxidants or exclusion of oxygen in the package.

## Data Availability Statement

The original contributions presented in the study are included in the article/supplementary material, further inquiries can be directed to the corresponding author/s.

## Author Contributions

NR and SG performed the research, analyzed the data, and wrote the original manuscript. NB provided technical support in data analysis. LM, XY, and BZ edited the manuscript. SB supervised the research design and reviewed the manuscript. All authors contributed to and approved the final draft of the manuscript.

## Funding

This work was supported by Chair Professor Grant (P-20-52297). Thailand's Education Hub for Southern Region of ASEAN Countries (TEH-AC, 2018) scholarship and Prachayajarn Scholarship, Prince of Songkla University (Grant No. AGR6402088N) are also acknowledged.

## Conflict of Interest

The authors declare that the research was conducted in the absence of any commercial or financial relationships that could be construed as a potential conflict of interest. The handling editor declared a past co-authorship with one of the authors, SB.

## Publisher's Note

All claims expressed in this article are solely those of the authors and do not necessarily represent those of their affiliated organizations, or those of the publisher, the editors and the reviewers. Any product that may be evaluated in this article, or claim that may be made by its manufacturer, is not guaranteed or endorsed by the publisher.
